# Determinants of antibiotic prescription in children with adenovirus respiratory tract infections

**DOI:** 10.1007/s00431-024-05615-2

**Published:** 2024-05-23

**Authors:** Danilo Buonsenso, Anna Camporesi, Francesca Viozzi, Ilaria Lazzareschi, Lavinia Manca, Annamaria Caci, Daniele Menna, Rosaria Santangelo, Maurizio Sanguinetti, Francesca Raffaelli, Barbara Fiori, Giuseppe Zampino, Piero Valentini

**Affiliations:** 1 Department of Woman and Child Health, Fondazione Policlinico Universitario A. Gemelli IRCCS, Largo A. Gemelli 8, 00168 Rome, Italy; 2 Centro di Salute Globale, Università Cattolica del Sacro Cuore, Rome, Italy; 3 Anesthesia and Intensive Care Unit, ‘Vittore Buzzi’ Children’s Hospital, Milan, Italy; 4 Medicine and Surgery, Università Cattolica del Sacro Cuore, Rome, Italy; 5 Dipartimento di Scienze di Laboratorio e Infettivologiche, Fondazione Policlinico Universitario A. Gemelli IRCCS, 00168 Rome, Italy; 6 Dipartimento di Scienze Biotecnologiche di Base, Cliniche Intensivologiche e Perioperatorie-Sezione di Microbiologia, Università Cattolica del Sacro Cuore, 00168 Rome, Italy

**Keywords:** Adenovirus, Antibiotics, Children

## Abstract

We performed this study to evaluate factors associated with antibiotic prescriptions in children with adenovirus infection, since no studies have attempted to address this aspect in the pediatric population. Retrospective study of children younger than 18 years of age tested positive for adenovirus on a syndromic nasopharyngeal test from 2018 to 2023. We compared the need of pediatric intensive care unit (PICU), invasive ventilation, and other respiratory support, viral etiologies, clinical presentations, imaging, and laboratory results in the precovid (2018–2019) and covid (2020–2022) period. The use of antibiotics was studied with multivariable logistic regression including demographic as well as clinical data as covariates. Two hundred fifty-eight patients were enrolled. One hundred fifty-eight patients received an antibiotic (mean duration 6.2 (±2.7) days (median 4; IQR: 4–7)). Presence of seizures and C-reactive protein values as predictors for antibiotic prescription (OR for seizures: 12.17; 95% CI: 1.42–103.91; *p* = 0.022; OR for CrP: 1.03; 95% CI: 1.01–1.04; *p* = 0.001). Seventy-four patients received intravenous antibiotics (74/156, 47.4%). Risk factors for intravenous antibiotic were the presence of decay (OR: 3.74; 95% CI: 1.25–11.71; *p* = 0.018), CrP values (OR: 1.02; 95% CI: 1.00–1.03; *p* = 0.001), and presence of seizures (OR: 16.34; 95% CI: 2.65–100.83; *p* = 0.003). Duration of intravenous antibiotics correlated with the presence of seizures (Coeff: 1.6; 95% CI: 0.41–2.89; *p* = 0.009) even when adjusted for CrP values.

*    Conclusion*: The clinical presentation of adenovirus infection in children is non-specific, leading to frequent antibiotic prescription despite bacterial co-infections was rare. Higher CrP values and presenting with seizures are significantly associated with a higher risk of receiving antibiotics. Rapid microbiological tests and newer biomarkers can help clinicians to improve antibiotic prescription in this cohort of children.

**Table:** 

**What is Known:** **•** *Adenovirus infection is a common cause of fever and respiratory tract infections in children.* *•* *Children with adenovirus infections frequently receive antibiotics, but determinants of this practice are poorly established.*
**What is New:** **•*** Higher C-reactive protein values and presenting with seizures are significantly associated with antibiotic prescription.* • *Since the beginning of COVID-19 and implementation of rapid diagnostics, less children with adenovirus infection received antibiotics.*

**Supplementary information:**

The online version contains supplementary material available at 10.1007/s00431-024-05615-2.

## Introduction

Adenoviruses (HAdVs) are non-enveloped, double-stranded linear DNA viruses. Currently, 116 different HAdVs are known, classified into seven species A to G [1]. Adenoviruses have been isolated in the upper respiratory tracts and gastrointestinal systems of humans and transmission happens through droplets and contact with infected secretions or stools [2]. As such, HAdVs are particularly frequent in children younger than 5 years since they spend most of their daily time in closed environments, such as daycare centers, orphanages, or other institutions. Although respiratory infections are the most frequent disease caused by HAdVs in children, several other systems can be affected, including the central nervous system, the gastrointestinal and urinary tract, eyes, and cardiovascular system. Different types of HAdV have been linked with a specific clinical manifestation, suggesting that they may have different pathogenic properties and tissue tropism [3]. For example, HAdV 2-3-4-7-14 are frequently associated with pharyngoconjunctival fever, HAdV 2-3-5-7-31-40-41 with gastrointestinal symptoms, HAdV-7 with central nervous system involvement, HAdV 1-3-5-7 with upper respiratory and HAdV 3-4-7-21 with lower respiratory tract symptoms [1–3].

While our understanding of HAdV infection in children has significantly increased in the recent decades, its complete understanding and epidemiological picture are not yet completely known, also because HAdV is the most probably underdiagnosed, due to the self-limiting presentation (leading to testing only most severe cases) but also the low sensitivity of traditional tests like direct immunofluorescence antibody [4]. While characteristic clinical presentations can be clinically suspected (e.g., in the case of pharyngoconjunctival fever), several other manifestations, including respiratory infections, can be clinically indistinguishable from other viral infections, and only be recognized in the contest of syndromic testing with multiplex panels on specific materials (e.g., nasopharyngeal swabs or stools) [1, 3]. However, such tests have only recently been implemented and not all centers have routine access to these diagnostics, therefore limiting our complete understanding of the full picture of HAdVs in children. In addition, as HAdVs may be a relatively common cause of high fever and respiratory symptoms, it is plausible to hypothesize that this viral infection accounts for a relatively large number of potentially inappropriate antibiotic prescriptions. To our knowledge, only one study published in 2004 on 143 HAdV cases documented that up to one third of children received antibiotics, despite only two had documented bacterial infection, suggesting that rapid tests to diagnose this disease can significantly impact antibiotic prescriptions [5].

In our institution, since 2018, syndromic panels for nasopharyngeal testing that include HAdV are available and in use for children evaluated for respiratory symptoms or fever of unknown origin, therefore allowing us to largely recognize HAdVs even in undifferentiating infectious syndromes. As such, we performed this study to further characterize the clinical presentations of HAdVs in children with signs and symptoms of respiratory tract infections (RTI) and to evaluate factors that are associated with antibiotic prescriptions in the context of this viral infection, therefore trying informing future practice to improve inappropriate antibiotic prescriptions.

## Materials and methods

### Study population

This is a retrospective study performed in children hospitalized upper or lower RTI and tested for respiratory viruses at the Agostino Gemelli University Hospital in Rome from January 2018 to November 2023. The study is part of a larger retrospective study investigating changes in children presenting with acute symptoms before and during the pandemic, approved by the Ethics Committee of our institution (ID 3497, Prot 0049226/20). The original study investigated how acute presentations in changed in Europe before and after the pandemic [6], with an updated cohort now under studying. Informed consent was not needed according to local Ethic Committee rules given the retrospective nature of the study using aggregate data.

For this study, we included all patients younger than 18 years of age with diagnosed with a clinical diagnosis of LRTI in our hospital (either in the emergency department or in the pediatric inpatient unit) and tested with a syndromic panel for respiratory viruses with nasopharyngeal swab, and turned out to be positive for HAdV. We only excluded children that were discharged directly from the emergency department without being tested with syndromic respiratory panels, or tested positive for viruses different than HAdV.

The primary aim of this study was to understand the burden of antibiotic prescriptions in children with microbiologically-confirmed HAdV and factors associated with a higher risk of receiving antibiotics in these children. Secondary outcomes were to characterize clinical presentation, laboratory markers, admission rates and length-of-stay, severity (measured as need of pediatric intensive care unit (PICU), and need of different respiratory support (in increasing order of severity, from no oxygen support, to low-flow oxygen, high-flow nasal cannulae (HFNC), continuous positive airway pressure (CPAP) and invasive ventilation).

Secondary aims were to describe the changing patterns of isolated viruses in hospitalized children.

### Kits for respiratory viruses

During the study period, two different kits have been used, as previously described [7]. For in-patient testing, the Allplex™ Respiratory Panels 1, 2 and 3 Seegene System is a molecular method for the genome detection and typing of the following respiratory viruses: respiratory syncytial virus (RSV A and B), influenza virus ((Flu A and B), parainfluenza viruses (PIV 1, 2, 3 and 4), adenovirus (AdV), enterovirus (HEV), metapneumovirus (MPV), rhinovirus (HRV), bocavirus (HBoV), and coronavirus (CoV NL63, 229E, OC43) in different types of clinical specimens.

In case of emergency testing (e.g., tests performed already in the emergency department), we used multitarget tests that require 45/60 min of time and can be performed directly from a primary clinical sample. Obviously, these tests have a lower sensitivity than the reference method that remains the multiplex Real-Time RT-PCR One-step (Allplex™ Seegene) which includes genome extraction and gene amplification with specific target primers. These options were used both in the pre and pandemic period when the test was requested directly from the pediatric emergency department. We used several panels:

- ePlex Respiratory Panel (RP).
- Later replaced with ePlex Respiratory Panel (RP2) which adds the Sars COV 2 virus to the previous version, i.e., RP.
- Film array Respiratory Panel 2.1.
- QIAstat Respiratory Panel^®^ assay (QIAstat RP).

The two settings of testing (for inpatients and emergency department settings) were both available in both periods (pre- and pandemic- periods) and, evaluating the same pattern of respiratory viruses (with the only exception of SARS-CoV-2, which was not considered in this study), their use should not have had any impact on respiratory virus detection changes before and during the pandemic. In terms of sensitivity, studies showed concordant performance of the AllPlex respiratory panels, QIAstat RP, Eplex, and the FilmArray panels for the simultaneous detection of multiple respiratory viruses [8–10].

For microbiologically confirmed bacterial co-infections, we referred to isolation of bacteria through traditional culture or molecular tests in sterile fluids (e.g., cerebrospinal fluid), or in clinically relevant specimens with compatible presentation (e.g., urine culture along with abnormalities in urine tests, or a throat swab that the attending physician decided to treat), or a clinical presentation strongly associated with a bacterial etiology, even in the absence of a microbiological confirmation (e.g., retropharyngeal abscess).

### Statistical analyses

Categorical variables were described as frequencies and percentages, and continuous variables were expressed as mean (±standard deviation) or median [interquartile ranges] as appropriate. Patients were also studied according to the two designated viral seasons: the pre-pandemic (1 January 2018–1 January 2020) and post-covid period (1 January 2020–31 November 2023).

We used the chi-square test or Fisher exact test as appropriate to analyze categorical variables and the Student’s *t*-test or Wilcoxon rank-sum test as appropriate for continuous variables. Blood laboratory exams were used as outcomes for multivariable linear regression models; the final models were chosen after checking that variance inflation factors of the variables included in the model did not exceed the threshold of 5 [11]. Use of antibiotics (prescription and choice of intravenous antibiotic) was studied with multivariable logistic regression including demographic as well as clinical data as covariates. Model was selected with a step-down method and final model was chosen according to the best Pseudo-R2. Goodness-of-fit (GOF) of the final models has been proved with the Hosmer-Lemeshow test. All statistical tests were two-sided and the level of statistical significance was set at 0.05. Data have been analyzed with Stata 18 BE (StataCorp. 2023. Stata Statistical Software: Release 18. College Station, TX: StataCorp LLC).

## Results

### Study population

Two hundred fifty-eight patients were enrolled in the study. Demographic characteristics and clinical presentation are presented in Table 1. A minority of children (40, 15.6%) presented comorbidities.

A microbiologically confirmed bacterial co-infection was documented in 19 children (7.3%). Further details about imaging findings, treatments performed, and main clinical outcomes are reported in Table 2. C-reactive protein (CrP) values were associated in multivariable linear regression with vomit and diarrhea as symptoms (Coeff: 14.7; 95% CI: 1.86–27.64; *p* = 0.025) and presence of physical decay, in this case with a negative relationship (Coeff: −13.79; 95% CI: −27.42 to −0.016; *p* = 0.047). Procalcitonin values did not show any significant relationship with symptoms. Alanine transferase (ALT) values were associated with presence of vomit/diarrhea (Coeff: 31.94; 95% CI: 5.44–38.43; *p* = 0.018) and presence of metapneumovirus (Coeff: 155.90; 95% CI: 91.98–219.82; *p* > 0.001). AST levels were associated with metapneumovirus presence (Coeff: 220; 95% CI: 31.55–409.25; *p* < 0.001).

**Table 1:** Demographic information and clinical presentation of children with adenovirus infection

	**Total**
	***N*** **= 258**
**Demographic**	
Male sex	92 (35.7%)
Age (months)	15.0 (9.0–23.0)
Children younger than 90 days	9 (3.5%)
Comorbidities	40 (15.6%)
Heart disease	3 (1.2%)
Respiratory disease	7 (2.7%)
Metabolic/neuromuscular	10 (3.9%)
GI disease	7 (2.7%)
**Clinical presentation**	
Vomit/diarrhea	90 (34.9%)
Decay	53 (20.5%)
Respiratory difficulty	41 (15.9%)
Seizures	28 (11.0%)
Jugular recessions	12 (4.7%)
Thoracic recessions	11 (4.3%)
Diaphragmatic recessions	11 (4.3%)
Intercostal recession	9 (3.5%)
Nasal flare	1 (0.4%)
Respiratory evaluation	
RR, *n*/minute	33.5 (22.0–40.0)
Spo2, %	97.0 (95.0–98)
Crackles	31 (12.1%)
Bilateral crackles	25 (9.7%)
Reduced air entry	17 (6.6%)
Wheeze	15 (5.8%)
Bilateral wheeze	15 (5.8%)
Bilaterally reduced air entry	11 (4.3%)

*GI g*astrointestinal, *RR r*espiratory rate

**Table 2:** Laboratory and imaging findings, microbiological results, and treatments received by children with adenovirus infection

	**Total**
	***N*** **= 258**
**WBC**	12585 (8440–16060)
**Neutrophils**	6990 (4420–10180)
**Lymphocytes**	3820 (2680–5060)
**CrP, mg/L**	33.7 (15.2–67.2)
**PCT, ng/mL**	0.4 (0.18–1.44)
**AST**	16 (11–22)
**HB**	11.5 (10.7–12.3)
**Platelets**,	329000 (260000–429000)
**Chest X-ray**	69 (26.8%)
* Lung consolidations*	31 (12.1%)
* Interstitial pattern*	16 (6.2%)
* Atelectasis*	3 (1.2%)
* Pleural effusion*	2 (0.8%)
* Pneumothorax*	0 (0.0%)
**Viral swab positivity**	257 (99.6%)
* Influenza A/B*	3 (1.16%)
* RSV*	13 (5.04%)
* Enterovirus*	59 (22.87%)
* Parainfluenza*	14 (5.43%)
* Metapneumovirus*	7 (2.71%)
* Bocavirus*	16 (6.2%)
* Rinovirus*	80 (31.01%)
* Coronavirus*	13 (5.04%)
* SARS-CoV-2*	2 (0.78%)
**Viral co-infection**	121 (46.9%)
**Bacterial co-infections**	
Positive on urine culture * E. coli*	4 (1.6%) 4 (100%)
Positive on blood culture * S. typhi*	1 (0.4%) 1 (100%)
Positive on CSF culture	1 (0.4%)
Group A Strep pharyngitis	9 (3.5%)
Retropharyngeal abscess	1 (0.4%)
Bacterial enterocoloties (*E. coli* enteropathogen)	1 (0.4%)
Skin abscess (*S. aureus*)	1 (0.4%)
Spondylodiscitis	1 (0.4%)
**Treatments**	
Steroids	43 (16.7%)
Antibiotic	158/258 (61.24%)
Oral antibiotic only	74/156 (47.4%)
IV antibiotic only	43/156 (27.5%)
Antibiotic duration	7.0 (4.0–7.0)
Two antibiotics duration	6.0 (3.0–7.0)
Intravenous antibiotic duration	0.0 (0.0–3.5)
Two intravenous antibiotics duration	3.0 (0.0–6.0)
Intravenous fluids	86 (33.5%)
Oxygen	37 (14.3%)
Broncodilathors	35 (13.6%)
Low flow oxygen	23 (8.9%)
HFNC	15 (5.8%)
CPAP	3 (1.2%)
MV	5 (1.9%)
PICU	13 (5.1%)
PICU LOS	
Hospital LOS, days	0.0 (0.0–5.0)

*WBC* white blood cells, *HFNC* high flow nasal cannulae, *CPAP *continuous positive airway pressure, *MV *mechanical ventilation, *PICU *pediatric intensive care unit, *LOS *length of stay

### Determinants of antibiotic prescription

One hundred fifty-eight patients received an antibiotic. The mean duration of antibiotic therapy was 6.2 (±2.7) days (median 4; IQR: 4–7). The most frequently used antibiotics were amoxicillin (8), amoxicillin-clavulanic acid (52), cefixime (12), clarithromycin (4), cefaclor (1), ceftriaxone (32), piperacillin/tazobactam (1), cefpodoxime (1), and amikacin (1).

**Fig. 1:**
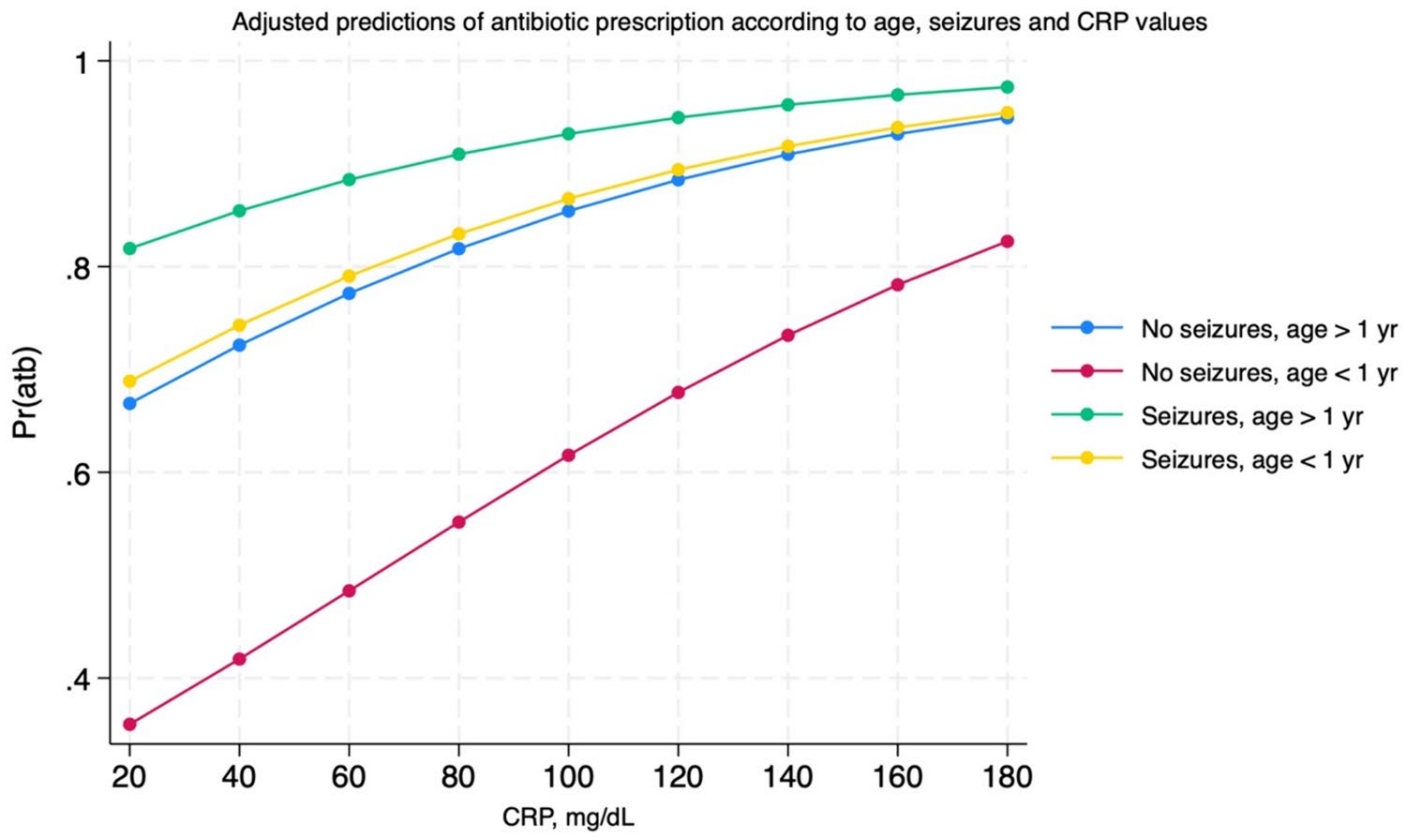
Relationship between age, neurological symptoms, and CrP values with antibiotic prescription in children with adenovirus infection

The best multivariable logistic regression model conducted on the use of antibiotics showed the presence of seizures and CrP values as predictors for it (Table 3). Age was close to significance with a trend towards more antibiotic prescriptions in older patients. If we divide patients into two categories (older vs. younger than 1 year) being younger is a protective factor against antibiotic use. The relationship between age, neurological symptoms, and CrP are illustrated in Fig. 1.

Duration of antibiotic therapy was associated with seizures with a negative relationship (Coeff: −1.53; 95% CI: −2.73–033; *p* = 0.013).

**Table 3:** Multivariable logistic regression for the outcome “antibiotic prescription”

**Antibiotic prescription**	**Odds ratio**	***P*** **> z**	**[95% conf. interval]**	
Age (months)	1.04	0.089	0.99	1.1
Seizures	12.49	0.023	1.41	110.47
CrP, mg/dl (deciles)	1.45	0	1.19	1.77

Pseudo-R2: 0.63. G-O-F: Prob > chi2 = 0.44

Subgroups of patients under 3 months and under 1 month of age were further analyzed in the relationship with antibiotics; both subgroups showed no significant relationship with antibiotic prescription (Fisher’s exact test for the group under one month: *p* = 0.379; for the group under 3 months: *p* = 0.259). The previous logistic model was repeated substituting the age variable with a binary variable—age under one month or age under three months—and yielded the same results.

Seventy-four patients among those receiving antibiotics (74/156, 47.4%) received an intravenous antibiotic. In 43 cases this was the only antibiotic, in the remaining also an oral antibiotic had been prescribed.

Risk factors for intravenous antibiotic course were the presence of decay, CrP values, and presence of seizures (Table 4). Duration of intravenous antibiotics correlated with presence of seizures (Coeff: 1.6; 95% CI: 0.41–2.89; *p* = 0.009) even when adjusted for CrP values.

Twenty-two patients received also a second antibiotic course, with a mean duration of 6.27 (±3.29) days (median 6; IQR: 4–7). Prescription of a second antibiotic course, adjusted for CrP or procalcitonin (PCT) values, was associated with lung consolidation (OR: 8.4; 95% CI: 2.99–23.57; *p* < 0.001) and the presence of an interstitial pattern at CXR (OR: 11.59; 95% CI: 3.38–39.76; *p* < 0.001).

**Table 4:** Multivariable logistic regression for the outcome “use of intravenous antibiotics”

**Use of intravenous antibiotic**	**Odds ratio**	***P*** **> z**	**[95% conf. interval]**	
Decay	3.23	0.028	1.13	9.24
CrP, mg/L	1.27	0.006	1.07	1.52
Seizures	12.91	0.004	2.31	7.19
Age (months)	1.01	0.413	0.97	1.06

Pseudo-R2: 0.21. GOF: Prob > chi2 = 0.37. CrP values have been categorized into deciles

As shown in the Supplementary Material, in the post-covid period, despite clinical presentation and demographic being similar, less children with HAdV received antibiotics.

## Discussion

To our knowledge, this is the largest series of children with microbiologically confirmed HAdV respiratory tract infections, showing that a significant proportion of these children, even without comorbidities receives antibiotics. About clinical presentation, our findings are in line with HAdV being a common cause of upper and lower RTI in children, estimated to cause 2–5% of pediatric respiratory tract infections and 4–10% of all pediatric cases of pneumonia [12]. However, our paper further confirmed that HAdV is also a common cause of febrile seizures, since in a large previous report it was described as a rare complication [5], but commoner in others [13–15].

In a previous report of HAdV in the USA, 46% of HAdV-positive children were given antibiotics at presentation, despite only 2 (1.4%) had documented bacterial infection (one had *Escherichia coli* urinary tract infection and one had *Moraxella catarrhalis* bacteremia) [5]. Thirty-six percent of children had a change in management based on positive point-of-care tests that were available, in that setting, in about 4 h, suggesting that rapid microbiological recognition of HAdV may positively impact care of patients and suggest the clinician that the patient has a viral infection. In particular, the most common change in management observed was the discontinuation of antibiotics as a result of the diagnosis of HAdV, which is a relevant finding considering the emerging threat of antibiotic resistance in the pediatric population and the frequency of HAdV in childhood. Unfortunately, in our study we were not able to systematically address after how long clinicians had viral results available and if these results contributed to stop antibiotics. However, since the pandemic, our microbiology implemented the capacity for viral testing which were more rapidly available, and it is of interest to note that in the post-covid period less children with HAdV received antibiotics, although we could not document a cause-effect relationship.

Importantly, the study from Rocholl et al. [5] was not able to define predictors on antibiotic use in children with HAdV infection, a data that can be useful in order to understand what potential areas of improvement in the management of these children are. Conversely, in our study, we were able to determine, through multivariable logistic regression model conducted on use of antibiotics, that the presence of seizures and CrP were predictors for antibiotic prescription, but also intravenous antibiotic. All together, these findings suggest that laboratory tests that are routinely available, like CrP, are not helpful for clinical practice to guide antibiotic prescriptions in children with viral illnesses where HAdV is a possible diagnosis, but that a combination of rapid viral respiratory testing and appropriate blood biomarkers can improve clinical practice. In fact, previous studies showed that HAdV can present with high CrP values [16–20] and, although the evidence is less straightforward, also high PCT values [21]. In a recent systematic review, we also showed that both CrP and PCT may wrongly classify HAdV infections as bacterial ones [22]. Recent in vitro study has confirmed that the exposure to HAdV induces an immediate and relevant innate host response inducing the release of acute-phase proteins, inducing in alveolar macrophages and peripheral blood mononuclear cells the expression of mRNA of IL-6 and TNF-α [23, 24]. As such, children with HAdV infections frequently receive antibiotics, as routine inflammatory markers usually mimic bacterial infections and rapid viral tests are not available in routine settings. Even when a microbiological evidence of HAdV is available, since the clinical and biomarker presentation can be similar for adenoviral and adenoviral-bacterial co-infection, it is challenging to confidently diagnose adenoviral infection [19], as adenovirus detection neither establishes active infection [25, 26] nor excludes the possibility of a bacterial co-infection [27]. In fact, HAdV can be detected in asymptomatic children [25, 26] and establish persistent/latent infection [28]. Thus, the initial decision not to treat with antibiotics cannot be based on PCR results alone. Our results are in line with evidence, showing that CrP levels and febrile seizures are strongly associated with antibiotic use, including intravenous ones. Therefore, our study reinforces the need of biomarkers that are able to better identify children with HAdV that would not benefit from antibiotics. In this regard, Stein et al. found that a biosignature based on CrP, TRAIL and IP-10 accurately (and better than other markers) differentiated between adenoviral and bacterial-adenoviral infection in this cohort of PCR-positive adenovirus patients, even when inflammatory markers were high. The diagnostic accuracy of BV was extrapolated to represent a potential reduction in antibiotic overuse of 1.6-fold, from 24.3 to 14.8%, with no significant impact on antibiotic underuse [29].

Of note, after the beginning of the pandemic, the proportion of children with adenovirus infection that did not receive antibiotics increased (data presented in Fig. 5s). Our hypothesis is that, since the pandemic, our center has obtained more experience in the interpretation of viral testing (as we reviewed in a recent study analyzing antibiotic stewardship programs in the emergency department [30], being more prone in not routinely using antibiotic in these patients as presented in a flowchart that we published elsewhere [31].

Interestingly, we found that children under 1 year of age were at lower risk of receiving antibiotics. This was a relatively unexpected finding, since febrile young infants traditionally receive more frequently empirical antibiotic therapy. This historical approach more frequently refers mostly to children under 90 days of life [32]. However, the most recent guidelines of the American Academy of Pediatrics have changed this approach, and currently included recommendations for safe withholding of empirical antibiotics in well appearing infants with normal inflammatory markers, even in the age range of 21 to 90 days of life [33]. In our cohort, of the children younger than 1 year of age, only 9 were younger than 90 days of life, and this may explain why children < 12 months old received less antibiotics, in addition to the application in our Pediatric departments of the latest AAP guidelines, along with other antibiotic stewardship practices based on viral testing [31].

Our study is not without limitation. The retrospective nature is an intrinsic limitation. Secondly, although all children had signs and symptoms compatible with viral infections (as they were tested with syndromic panels by the attending physicians), we could not discriminate between HAdV colonization and real infection. Third, we were not able to collect information about after how long results from viral testing were available and how these tests contributed to earlier stop of antibiotic prescription. Fifth, our laboratory does not provide us with the specific adenovirus serotype, therefore we could not address if this factor is somehow associated with a higher risk of bacterial infection. Last, antibiotic prescriptions were decided by the assessing clinician, which may be biased but still represents real-life settings. Strength of our paper is the largest study that provided a detailed clinical presentation and factors associated with antibiotic prescription in children with HAdV.

In conclusion, our study showed that HAdV infection in children causes a variety of clinical syndromes and a significant proportion of children with HAdV receive antibiotics, including broad-spectrum and intravenous ones. Higher CrP values and presenting with seizures are significantly associated with a higher risk of receiving antibiotics. Future studies should prospectively investigate strategies to reduce the prescription, and duration, of antibiotics in children with suspected or confirmed HAdV. In this context, rapid microbiological tests and newer biomarkers can help clinicians to improve antibiotic prescription in children with HAdV. Also, studies using data from large multicenter cohorts analyzed with modern artificial intelligence tools may help the development of predicting clinical tools of probable HAdV infection in children, therefore limiting the use of antibiotics.

### Supplementary Information

Below is the link to the electronic supplementary material.

Supplementary file1 (DOCX 828 kb)

## Data Availability

Available upon request to the corresponding author.
