# Supplementary material for: Determinants of antibiotic prescription in children with adenovirus respiratory tract infections
Source: Eur J Pediatr. 2024 May 23;183(8):3489–97. doi: 10.1007/s00431-024-05615-2 (PMC11263461; doi:10.1007/s00431-024-05615-2)

Supplementary material

Before and after the beginning of the pandemic, children seen in hospital settings for adenovirus infection were more frequently with comorbidities (figure 1s).


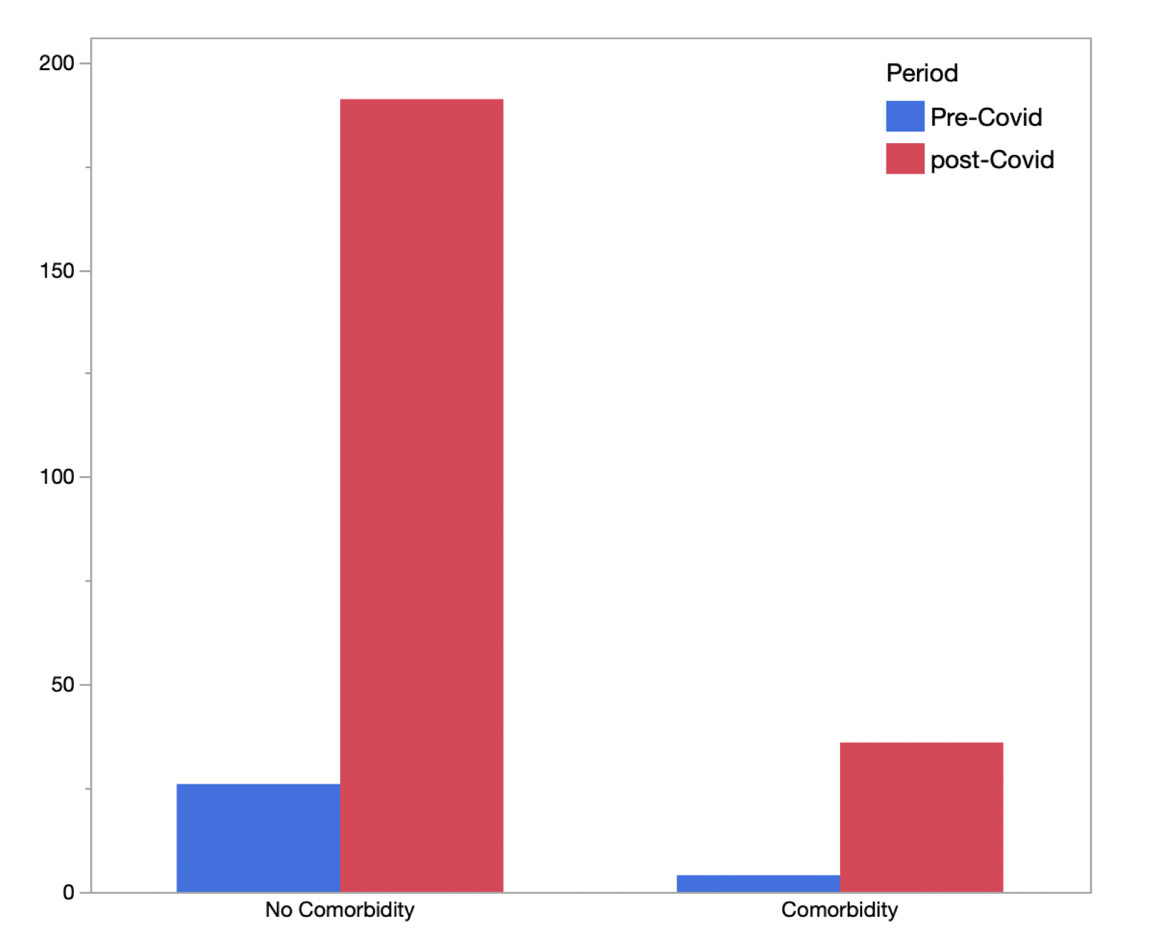


Age of patients was similar in the two study periods (figure 2s)


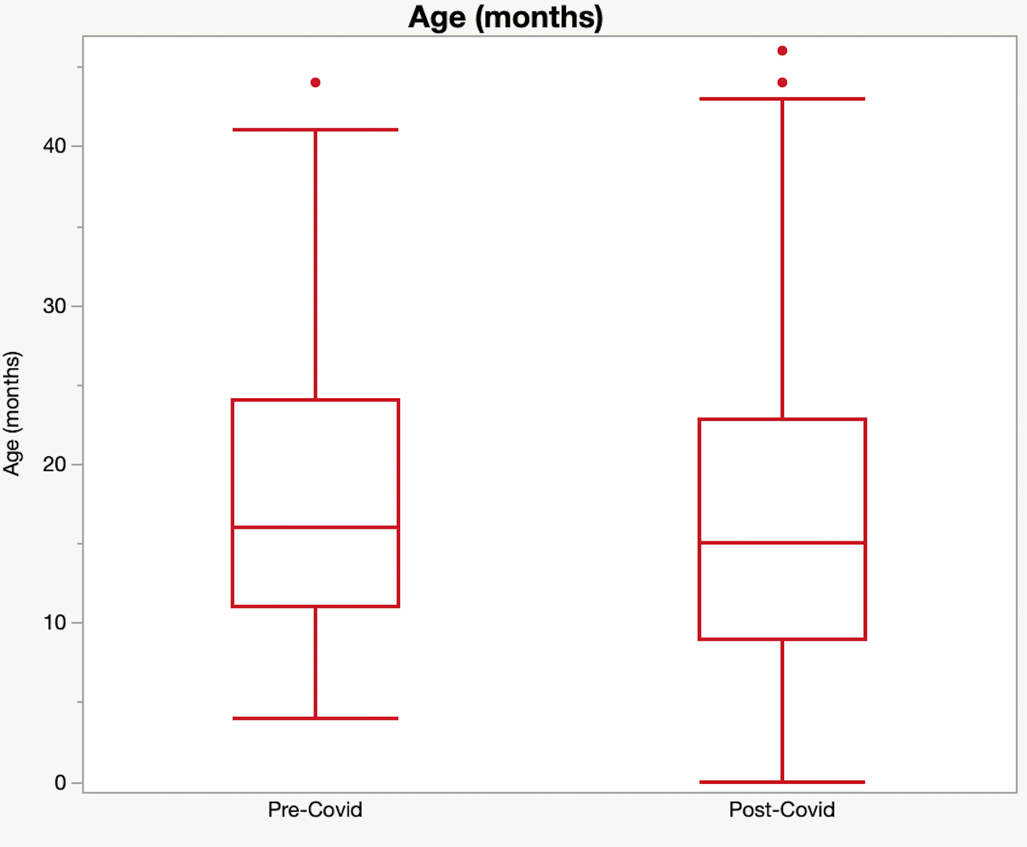


Also symptoms duration was similar before and after Covid (figure 3S).


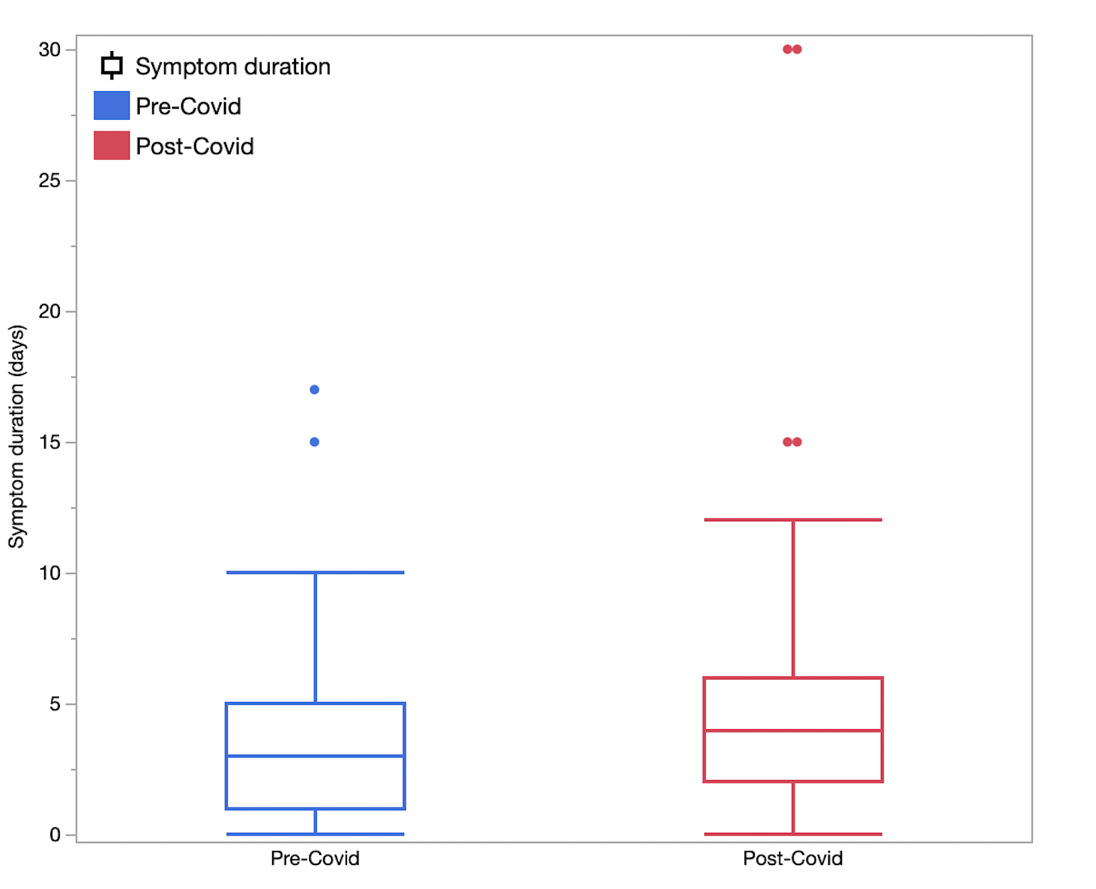


Less children since the beginning of the pandemic presented with seizures (figure 4s)


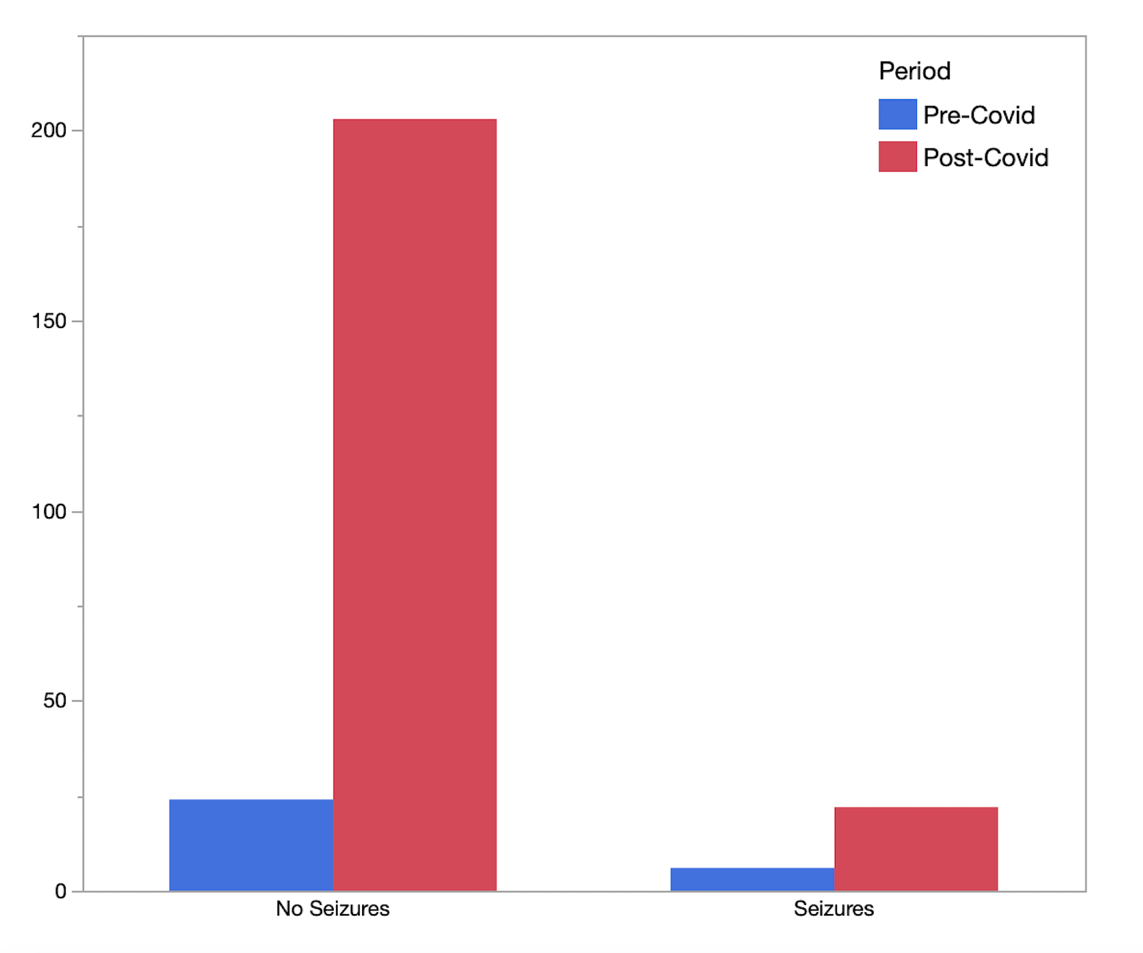


After the beginning of the pandemic, the proportion of children with adenovirus infection that did not receive antibiotics increased (figure 5s). Our hypothesis is that, since the pandemic, our center has obtained more experience in the interpretation of viral testing, being more prone in not routinely using antibiotic in these patients as presented in a flowchart that we published elsewhere (31).


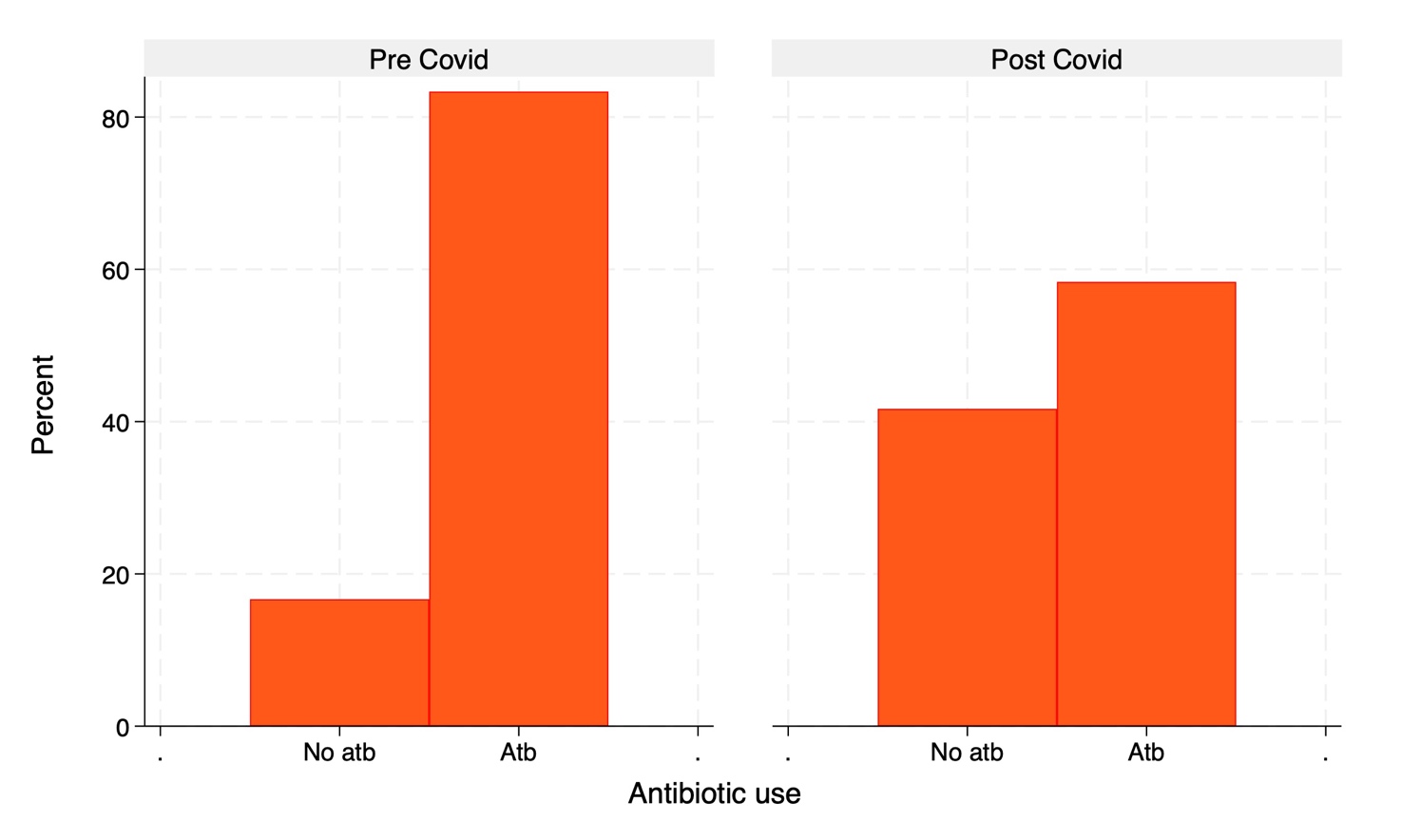

Supplement: Supplementary file 1 — Supplementary file1 (DOCX 828 kb) [file 431_2024_5615_MOESM1_ESM.docx]
